# Correlation between mitral valve area and left atrial function in rheumatic mitral valve stenosis patients

**DOI:** 10.5830/CVJA-2022-059

**Published:** 2022-12-13

**Authors:** Ahmed Fareed, Fathy Makaldy, Omar Saleh, Mohamed Hamed

**Affiliations:** Department of Cardiology, Suez Canal University, Ismailia, Egypt; Department of Cardiology, Ismailia General Hospital, Ismailia, Egypt

**Keywords:** rheumatic heart disease, mitral stenosis, mitral valve area, left atrial function, atrial fibrillation

## Abstract

**Background:**

Rheumatic heart disease (RHD) continues to be one of the leading causes of cardiovascular morbidity and mortality. The mitral valve frequently develops mitral stenosis (MS), and it is the most prevalent valve lesion in patients with chronic RHD. Left atrial (LA) functional impairment is associated with rheumatic MS.

**Aim:**

The aim of this study was to evaluate the association between LA function and mitral valve area (MVA) in rheumatic MS patients, and to assess the echocardiographic parameters in sinus rhythm and atrial fibrillation (AF) patients.

**Methods:**

This was a cross-sectional, descriptive study that involved patients with rheumatic MS. Patients underwent a standard 12-lead electrocardiogram and echocardiographic examination. MVA was assessed and correlated with LA function. Comparison was made between sinus rhythm and AF patients.

**Results:**

Eighty-one patients with rheumatic MS were included in this study, with 71.6% of them having associated MR. MVA showed a statistically highly significant positive correlation with LA and right ventricular (RV) function, and a statistically significant/highly significant negative correlation with their dimensions. A higher percentage of patients with severe MS was in AF (58.1%).

**Conclusion:**

There was a positive correlation between LA function and MVA in rheumatic MS patients. AF was related to the severity of MS.

Rheumatic heart disease (RHD) is a cardiac disease affecting the endocardium, myocardium and pericardium, with the subsequent potentiality of their permanent damage.^1^ RHD occurs as a result of rheumatic fever (RF), which is caused by group A β-haemolytic streptococcus infection.^2^ Developing countries show a high prevalence of RF due to overcrowding and poor sanitation.^3^

 Among the rheumatic cardiac complications, rheumatic mitral stenosis (MS) is characterised by a thickening of the mitral valve leaflets, commissural fusion and shortening and fusion of the chordae tendineae.^4^ This stenosis results in impeded left atrial (LA) to left ventricular (LV) blood flow, with subsequent blood stagnation in the left atrium. This stagnation causes elevation of pressure in the left atrium and pulmonary veins, pulmonary oedema and elevation of pressure in the pulmonary artery and the right side of the heart.^5^

 Atrial fibrillation (AF) is a common complication of rheumatic MS, which conveys a poor prognosis.^6^ AF is found in more than 80% of patients with MS and systemic embolism.^7^

 Rheumatic MS is mainly diagnosed by clinical examination and echocardiography.^8^ Echocardiography allows objective monitoring of the affected valve, the chamber size and function, and pulmonary artery pressure. It offers detailed data, which helps to determine the best management strategy.^9^

 RF is an ongoing problem in Egypt and is predominantly complicated by cardiac involvement. One recent study conducted in a large Egyptian cardiac centre announced that 46.1% of the cardiac operations performed in the centre during a period of 20 years were for RHD, and about 360 new RF/RHD cases were identified each year.^10^

 Despite these critical figures, few studies in Egypt evaluated the impact of mitral valve area (MVA) on LA function and AF prevalence in rheumatic MS patients. This study aimed to detect the relationship between LA function and MVA in rheumatic MS patients, and to assess the echocardiographic parameters in sinus rhythm patients and AF patients.

## Methods

This cross-sectional, descriptive study was performed after the approval of the regional ethics committee (No. 2019.6.11/3879) and in accordance with the Declaration of Helsinki on rheumatic MS patients who were referred for echocardiography in the Suez Canal University (SCU) Hospital during the period from July 2019 to September 2020. From each patient, written, informed consent was obtained.

 Adult patients with MVA < 2.5 cm^2^ and preserved LV systolic function were eligible for the study. Those excluded were LV systolic dysfunction patients (ejection fraction < 50%), those with ischaemic heart disease, more than moderate aortic valve disease, prosthetic aortic valves, sclerotic MS, electrocardiogram (ECG)- discovered atrioventricular (A-V) conduction abnormalities, and patients with low-quality echocardiographic images. All patients with MS referred for echocardiography in the SCU during the period from July 2019 to September 2020 and fulfilling the inclusion criteria were enrolled.

 Sample size was calculated according to:

 total sample size (n) = [(Zα + Zβ)/C]2 + 3

 where the standard normal deviation for α = Zα = 1.960, the standard normal deviation for β = Zβ = 0.842 and the expected correlation coefficient = 0.408.11 This yielded a sample size of 41 patients. However, we included 81 patients in this study.

 The included patients underwent detailed history taking through an interview using a predesigned anonymous questionnaire. The questionnaire required data concerning demographic characteristics, history of RHD and previous interventional surgery, and history of AF and other arrhythmias. Patients also underwent a general examination, including measurement of height and weight, and pulse rate. From height and weight, the body surface area (BSA) was estimated using Mosteller’s formula:12

$$\mathrm{BSA}\left(m^{2}\right)=\sqrt{\frac{\text{ height }(cm)\times \text{ weight }(kg)}{3600}}$$

The BSA was used as an index for the annular dimensions of the cardiac valves.^13^

 A standard 12-lead ECG was done to determine whether the patient was in sinus rhythm or AF, to detect any other arrhythmia, to assess ECG signs of chamber enlargement, such as p pulmonale, RV strain or right bundle branch block, and to identify A-V conduction abnormalities.

 The echocardiography examination and image acquisition were performed using the Acuson X300™ ultrasound system (Siemens, Germany). Images were interpreted and measures were taken using the Horos Mac 10.5.8 social advice image viewer system. The MVA was assessed in each patient.

 Assessment of MVA by planimetry, assessment of transmitral diastolic pressure gradient, pressure half-time (PHT) and systolic pulmonary artery pressure (SPAP) were performed according to Cherry et al.^14^

 An inferior vena cava (IVC) diameter of ≤ 2.1 cm that collapsed more than 50% with a sniff suggested a normal right atrial (RA) pressure of 3 mmHg (range 0–5 mmHg), while an IVC diameter of > 2.1 cm that collapsed less than 50% with a sniff suggested a high RA pressure of 15 mmHg (range 10–20 mmHg). An intermediate value of 8 mmHg (range 5–10 mmHg) was used in indeterminate cases in which the IVC diameter and collapse did not fit this paradigm.^15^

 MS severity was graded using the MVA, which was considered normal if it measured 4–5 cm^2^. An MVA of > 1.5, 1.5–1 and < 1 cm^2^ were interpreted as mild, moderate and severe grades of stenosis, respectively.^14^

 Severity of mitral regurgitation (MR) was assessed using the vena contracta (the area where blood goes through the valve leaflets). Because the breadth of the vena contracta correlates with the diameter of the regurgitant orifice area, it is a reliable indicator of the severity of MR. Less than 3 mm of vena contracta width was considered mild MR, while a width of 3–6.9 mm was considered moderate MR and ≥ 7 mm was considered severe MR.^16^

 The LA anteroposterior diameter was determined in the parasternal long-axis view perpendicular to the posterior wall long axis, leading edge to leading edge (M-mode) or inner edge to inner edge (2DE), and measured at the level of the aortic sinuses.^17^

 LA volume was assessed by the biplane area–length method, which is better at assessing the true LA area in cases of MS. This is because in MS there is a tented area below the mitral valve due to the deformation and doming of the leaflets. Other methods of calculating the LA area draw a straight line between the annulus and may miss this tented area.

 The biplane area–length approach was used at the end of systole to gather measurements from the frame immediately preceding the mitral valve opening. Apical four- and two-chamber (A4CH, A2CH) views were adjusted to be of good quality with no foreshortening. The endocardial border was enabled to be visualised and the maximal area was measured with a planimeter, excluding the area under the MVA, pulmonary veins and LA appendage. The length between the mid-line of the plane of the mitral annulus and the opposite superior aspect of the LA was measured.^17^

 The LA volume was computed in this study using the formula:

 LA volume = 0.85 (A4CH–A2CH)/L,

 where L is the average of the two lengths. As a qualitycontrol measure, we frequently examined for LA shortening and confirmed that the two lengths did not differ by more than 5 mm. A discrepancy of > 5 mm between the two lengths indicates that the LA may have foreshortened the lengths from one view; the lengths were then re-measured to guarantee accuracy. Moreover, LA volume index (LAVI) was calculated according to this formula:

 LAVI = LA volume/BSA.^18^

 To calculate LA function, the following formulae were used:

 LA total emptying volume = LAVmax – LAVmin,

 LA total emptying fraction = (LAVmax – LAVmin)/LAVmax.

 To obtain the LA maximum volume (LAVmax), the LA volume was measured at end-systole, just before the opening of the mitral valve (at the end of the T wave on the ECG), while for minimum LA volume (LAVmin), at end-diastole, the LA volume was measured just before mitral valve closure (at the beginning of the QRS complex on the ECG).^17^

 The ranges and severity cut-off values for LA area and volume were interpreted in accordance with the American Society of Echocardiography and European Association of Cardiovascular Imaging Standards for quantifying cardiac chambers in adults using echocardiography.^17^

 The primary outcome of the study was the potential correlation between MVA and LA function, whereas the secondary outcome was the differences in echocardiographic parameters of LA function between patients in sinus rhythm and AF.

## Statistical analysis

The data were analysed by the statistical package for the social sciences (version 25.0; SPSS Inc, Chicago, Illinois, USA) software for Windows. Continuous variables are expressed as mean ± standard deviation (SD) and categorical variables are expressed as absolute numbers and percentage. The Spearman correlation test was used to measure the correlation between quantitative variables. Comparisons of continuous variables were performed using the unpaired Student’s t-test and comparisons of categorical variables were performed using the chi-squared test. A p-value < 0.05 was considered statistically significant.

## Results

This study included 81 patients with rheumatic MS, and of these, 58 had associated MR. The mean age of the patients was 47.40 ± 12.30 years and showed female predominance (females constituted 79% of the study patients). The patients’ mean BSA was 1.87 ± 0.20 m^2^ and mean heart rate was 78.52 ± 15.03 beats per minute. The majority of the patients (about 76.5%) were newly diagnosed with RHD (Table 1).

**Table 1:** Echocardiography measurements of the study patients (n = 81)

*Variables*	*Values*
itral valve, mean + SD	
MVA plan (cm²)	1.29 + 0.48
MPG (mmHg)	10.38 + 6.25
PHT	183.4 + 62.85
PASP (mm Hg)	46.78 + 15.81
R grades, n (%)	
Absent	2 (2.5)
I	23 (28.4)
II	44 (45.3)
III	9 (11.1)
IV	3 (3.7)
R grades, n (%)	
Absent	23 (28.4)
Grade I	10 (12.3)
Grade II	26 (32.1)
Grade III	16 (19.8)
Grade IV	6 (7.4)
V measurements, mean + SD	
EF %	63.28 + 5.12
IVSd (ml)	9.05 + 1.54
LVPWd (ml)	8.95 + 1.40
LVESD (ml)	31.49 + 4.06
LVEDD (ml)	48.75 + 4.91
LVMI (g/m²)	82.09 + 19.93
RWT (cm)	0.37 + 0.07
V measurements, mean + SD	
TAPSE (mm)	19.07 + 2.98
RVD (ml)	32.99 + 4.98
A area, mean + SD	
LAD (ml)	51.42 + 7.8
LAA S4V (ml)	29.86 + 8.32
LAA S2V(ml)	25.18 + 7.77
AvLS (ml)	6.40 + 0.94
LAA D4V (ml)	23.84 + 8.76
LAA D2V (ml)	19.90 + 7.90
avLD (ml)	5.86 + 1.05
A volume, mean + SD	
LAvol... (ml) "max	101.2 + 42.78
LAvol (ml)	71.01 + 39.84
LAVI. max (ml/m²)	54.32 + 23.9
LAVImin (ml m²	38.04 + 21.8
A function (%)	33 + 15

MVA plan: mitral valve area measured using planimetry, MPG: mean pressure gradient, PHT: pressure half-time, SPAP: pulmonary systolic pressure,

MR: mitral regurgitation, TR: tricuspid regurgitation, EF: ejection fraction, IVSd: interventricular septum diameter, LVPWd: left ventricular posterior wall diameter, LVESD: left ventricular end-systolic diameter, LVEDD: left ventricular end-diastolic diameter, LVMI: left ventricular mass index, RWT: relative wall thickness, TAPSE: tricuspid annular plane systolic excursion, RVD: right ventricular diameter, LAD: left atrial diameter, LAA S4V: left atrial area systolic four shaper view, LAA S2V: left atrial area systolic two shaper view,

LAA D4V: left atrial area diastolic four shaper view, LAA D2V: left atrial area diastolic two shaper view, LAvolmax: maximum left atrial volume, LAvolmin: minimum left atrial volume, LA function: left atrial function. AvLS: average length of systole, avLD: average length of diastole, LAVI: left atrial volume index.

 The echocardiography measurements of the patients are presented in Table 1. The mean MVA was 1.29 ± 0.48 cm^2^, and the most frequent tricuspid and mitral regurgitation grade was grade II (45.3 and 32.1% of patients, respectively). The patients’ mean ejection fraction was 63.28 ± 5.12%, mean RV function by tricuspid annular plane systolic excursion (TAPSE) was 19.07 ± 2.98 mm, while the mean LA function was 33 ± 15% (Table 1).

 According to echocardiographic assessment of valve stenosis, it was shown that 30% of cases had mild MS, 32% had moderate MS and 38% had severe MS. Comparison among patients with different grades of MS (mild, moderate and severe) with regard to the demographic data showed statistically significant differences in the gender distribution only (p = 0.002). Concerning echocardiographic findings, statistically significant/ highly significant differences were found in tricuspid regurgitation (TR) grades, and RV and LA measurements (Table 2).

**Table 2:** Demographic and echocardiographic characteristics of the studied patients according to the different MS grades

*Variables*	*Mild MS (n=24)*	*Moderate MS (n 26)*	*Severe MS (n=31)*	p-value
Age (years), mean + SD	48 + 10.99	46.54 + 11.85	47.65 + 13.89	0.93
Gender, n (%)				
Male	1 (4.2)	5 (19.2)	11 (35.5)	0.002b
Female	(95.8)	21 (80.8)	0 (64.5)	
BSA, mean + SD	1.88 + 0.17	1.87 + 0.24	1.88 + 0.20	0.94
Tricuspid regurgitation				
Normal morphology	24 (100)	25 (96.2)	29 (9.3)	0.38F
Rheumatic TR	0 (0)	1 (3.8)	3 (9.7)	
TR grades, n (%)				
Absent	2 (8.3)	0	0	0.01F
I	12 (50)	7 (26.9)	4 (12.9)	
II	10 (41.7)	15 (57.7)	19 (61.3)	
III	0	3 (11.5)	6 (19.4)	
IV	0	1 (3.8)	2 (6.5)	
LV measurements, mean	+ SD			
EF %	64.25 + 5.30	63.62 + 4.44	62.26 + 5.49	0.46
IVSd (ml)	9.21 + 1.77	8.96 + 1.4	9.00 + 1.51	0.87
LVPWd (ml)	8.96 + 1.57	8.73 + 1.28	9.13 + 1.38	0.54
LVESD (ml)	31+4.55	31.62 + 3.19	31.77 + 4.40	0.44
LVEDD (ml)	48.79 5.54	48.92 + 4.26	48.58 + 5.06	0.91
LVMI (g/m2)	32+21.63	81.65 + 18.85	82.52 + 20.09	0.85
RWT (cm)	0.38 + 0.07	0.36 + 0.05	0.38 + 0.08	0.60
RV measurements, mean	+ SD			
RVF (TAPSE) (mm)	20.13 + 3.94	19.31 + 2.84	18.06 + 1.77 ₽	0.001a
RVD (ml)	30.04 + 3.24	33.19 + 4.66	35.10 + 5.34	0.001
LA area, mean + SD				
LAD (ml)	46.79 + 7.27	52.38 + 7.71฿	54.19 + 6.9 P	0.001a
LA area S4 (ml)	25.45 + 9.27	30.77 + 8.03	32.50 + 6.43 €	0.001
LA area S2 (ml)	20.74 + 6.10	25.15 + 6.49	28.65 + 8.32 ₽	0.001a
AvLS (ml)	5.79 + 0.75	6.51 + 0.84 ฿	6.78 + 0.93	0.001a
LA area D4 (ml)	17.86 + 9.52	25.07 + 7.56 ฿	27.44 + 6.64 ₽	0.001a
LA area D2 (ml)	14.30 + 6.01	20.31 + 6.27 B	23.88 + 8.01 ฿	0.001a
avLD (ml)	5.02 + 0.82	6.02 + 0.91	6.37 + 0.95 ¹	0.001a
LA volume, mean + SD				
LAvolmax (ml)	80.03 + 46.46	101.33 + 35.03฿	117.45 + 39.6	<0.001a
LAvolmin (ml)	46.22 + 40.18	72.91 + 30.87B	88.61 +37.22	<0.001a
LAVI (ml/m2) "max	43.75 + 29.63	54.07 + 17.11B	62.71 + 21.1	<0.001
LAVI. min (ml/m2)	25.6 + 25	38.6 + 15B	47.2 + 19.5B	0.001a
LA function	0.47 + 0.14	0.29 + 0.14	0.26 + 0.10 B	<0.001a

BSA: body surface area, RHD: rheumatic heart disease, TR: tricuspid regurgitation, MS: mitral stenosis, EF: ejection fraction, IVSd: interventricular septum diameter, LVPWd: left ventricular posterior wall diameter, LVESD: left ventricular end-systolic diameter, LVEDD: left ventricular end-diastolic diameter , LVMI: left ventricular mass index, RWT: relative wall thickness, TAPSE: tricuspid annular plane systolic excursion, RVD: right ventricular diameter, LAD: left atrial diameter, LAA S4V: left atrial area systolic four shaper view, LAA S2V: left atrial area systolic two shaper view, LAA D4V: left atrial area diastolic four shaper view, LAA D2V: left atrial area diastolic two shaper view, LAvolmax: maximum left atrial volume, LAvolmin: minimum left atrial volume, LA function: left atrial function, AvLS: average length of systole, avLD: average length of diastole, LAVI: left atrial volume index. a*p*-values are based on the Kruskal–Wallis test, b*p*-values are based on the chi-squared test. Statistical significance at *p *< 0.05, F*p*-values are based on the Fisher exact test. Statistical significance at *p *< 0.05. Values with superscriptβ are different from mild MS group.

 MVA showed a statistically highly significant positive correlation with RV and LA function, and a statistically significant/highly significant negative correlation with RV and LA dimensions. Re-analysis of patients with isolated MS revealed the same significant association (Table 3).

**Table 3:** Correlation analysis between MVA and echocardiography parameters

	*MVA*	
*Variables*	*r*	*p-value*
Right ventricle		
TAPSE (mm)	0.472	0.000
RVD (ml)	-0.412	0.000
All study patients (n 81)		
LA area		
LAD (ml)	-0.473	< 0.001
LA area S4 (ml)	-0.415	<0.001
LA area S2 (ml)	-0.365	<0.001
AvLS (ml)	-0.435	<0.001
LA area D4 (ml)	-0.507	< 0.001
LA area D2 (ml)	-0.473	< 0.001
avLD (ml)	-0.530	< 0.001
LA volumes		
LAvolmax (	-0.383	<0.001
	-0.480	<0.001
LAVI.... (ml/m2)	-0.421	< <0.001
LAVI.... (ml/m²)	-0.512	< 0.001
LA function (%)	0.507	< 0.001
Isolated MS patients (n = 33)		
LA area		
LAD (ml)	-0.479	0.005
LA area S4 (ml)	-0.415	0.016
LA area S2 (ml)	-0.359	0.04
AvLS (ml)	-0.481	0.005
LA area D4(ml)	-0.511	0.002
LA area D2 (ml)	-0.426	0.013
avLD (ml)	-0.545	0.001
LA volume		
LAvolman(ml)	-0.418	0.047
LAvolmin (ml)	-0.507	0.014
LAVI... (ml/m²)	-0.410	0.018
LAVI... (ml/m²)	-0.506	0.003
LA function (%)	0.560	0.001

TAPSE: tricuspid annular plane systolic excursion, RVD: right ventricular diameter,

LAD: left atrial diameter, LAA S4V: left atrial area systolic four shaper view, LAA S2V: left atrial area systolic two shaper view, LAA D4V: left atrial area diastolic four shaper view, LAA D2V: left atrial area diastolic two shaper view, LAvolmax: maximum left atrial volume, LAvolmin: minimum left atrial volume, LA function: left atrial function. AvLS: average length systole, avLD: average length diastole, LAVI: left atrial volume index, *r*: Spearman’s correlation coefficient. *p-*values are based on Spearman’s correlation test as appropriate. Statistical significance at *p *< 0.05

 The ECG findings demonstrated that 47 patients were in sinus rhythm, while the remaining 34 were in AF. There was no statistically significant difference between patients in both categories in the distribution of different grades of MS severity (p = 0.06). Patients in AF had statistically significantly higher LA area and volume, and statistically significantly lower LA function (p = 0.003) (Table 4).

**Table 4:** Demographic and echocardiographic characteristics of the patients according to the different MS grades

*Variables*	*Sinus rhythm (n=47)*	*AF (n=34)*	*p-value*
MS severity			
Mild	17 (70.8)	(29.2)	0.06
Moderate	17 (65.4)	9 (34.6)	
Severe	13 (41.9)	18 (58.1)	
LA area			
LAD (ml)	49.7 + 6.83	53.79 + 8.6	0.026
LA area S4 (ml)	28.12 + 7.89	32.26 + 8.41	0.028
LA area S2 (ml)	22.84 + 6.08	28.43 + 8.73	0.003
AvLS (ml)	6.16 + 0.84	6.73 + 0.98	0.009
LA area D4 (ml)	21.54 + 7.82	27.02 + 9.10	0.005
LA area D2 (ml)	17.32 + 6.23	23.46 + 8.63	0.001
avLD (ml)	5.55 + 0.92	6.28 + 1.09	0.002
LA volumes			
LAVS (ml)	89.44 + 34.27	117.43 + 48.29	0.005
LAVD (ml)	58.53 + 31.13	88.27 + 44.35	0.001
LAVI.... (ml/m²)	47.9 + 18.2	63.1 + 27.96	0.008
LAVImin (ml/m²)	31.3 + 16.6	47.3 + 24.8	0.001
LA function	0.37 + 0.14	0.28 + 0.16	0.003

AF: atrial fibrillation, MS: mitral stenosis, EF: ejection fraction, IVSd: interventricular septum diameter, LVPWd: left ventricular posterior wall diameter, LVESD: left ventricular end systolic diameter, LVEDD: left ventricular end diastolic diameter, LVMI: left ventricular mass index, RWT: Relative wall thickness, TAPSE: tricuspid annular plane systolic excursion, RVD: right ventricular diameter, LAD: left atrial diameter, LAA S4V: left atrial area systolic four shaper view, LAA S2V: left atrial area systolic two shaper view, LAA D4V: left atrial area diastolic four shaper view, LAA D2V: left atrial area diastolic two shaper view, LAvolmax: maximum left atrial volume, LAvolmin: minimum left atrial volume, LA function: left atrial function. AvLS: average length systole, avLD: average length diastole, LAVI: left atrial volume index. a*p*-values are based on the Kruskal–Wallis test.

**Fig. 1:**
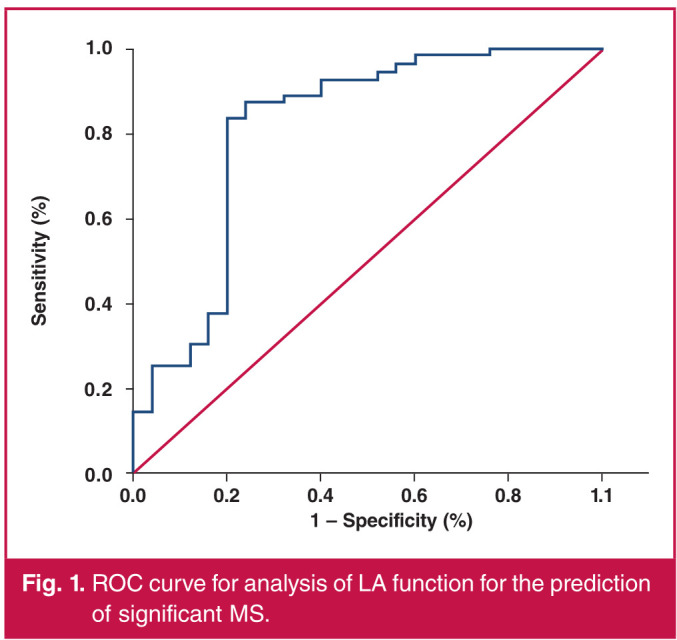
ROC curve for analysis of LA function for the prediction of significant MS.

 Receiver operating characteristic (ROC) curve analysis was performed for assessing LA function in predicting significant MS (moderate and severe MS) in RHD patients. The area under the curve (AUC) was 0.813, and a value of 39.4% was found to be the best cut-off point to predict significant MS among RHD patients, with a sensitivity of 83.93%, specificity of 80% and a statistically highly significant p-value (p < 0.001) (Fig. 1).

 Multivariate regression analysis was done to assess factors affecting the LA function. The model showed statistical significance and explained 87.9% of the variance determining LA function. LAVImin, LAVImax and MVA were the variables showing statistically significant association with LA function (p = 0.000).

**Fig. 2:**
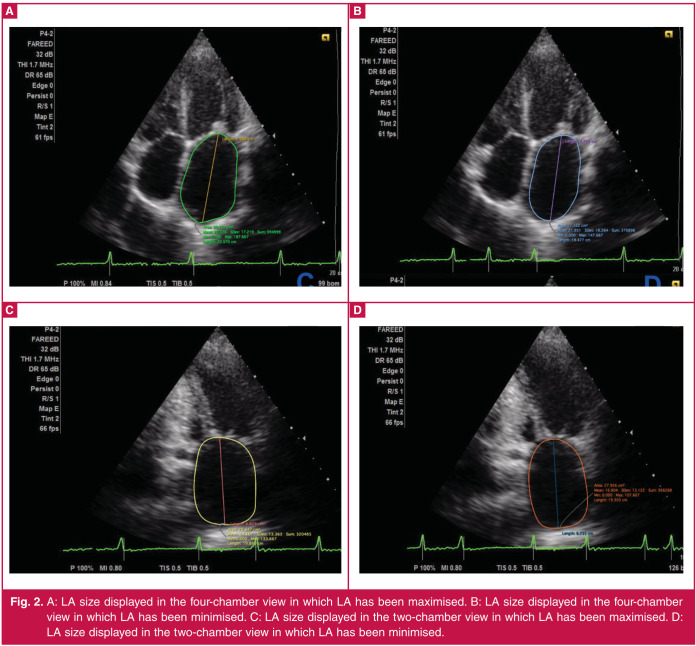
A: LA size displayed in the four-chamber view in which LA has been maximised. B: LA size displayed in the four-chamber view in which LA has been minimised. C: LA size displayed in the two-chamber view in which LA has been maximised. D: LA size displayed in the two-chamber view in which LA has been minimised.

 Two-dimensional (2D) echocardiography showing dynamic changes in LA area during the cardiac cycle, and an assessment of LA volume by the biplane area–length method in the patients are presented in Fig 2.

## Discussion

RHD is one of the most common causes of cardiovascular morbidity and mortality.^19^ The mitral valve is frequently involved in MS, and it is the most prevalent valve lesion in patients with chronic RHD.^20^ LA functional impairment has been reported to be associated with rheumatic MS. However, few studies have correlated LA function with the MVA.

 In our study, LA functional parameters were assessed by echocardiography and tested for possible correlation with the MVA. In addition, the differences between patients in sinus rhythm and AF concerning these parameters together with the MVA were evaluated.

 This study included 81 patients with rheumatic MS, with 71.6% of them having associated MR. The study patients had a mean age of 47.40 ± 12.30 years, and showed female predominance. Similarly, the study of Fu et al.^21^ reported that mixed MS and MR lesions were seen in 73.2 and 79.3% of their study groups with rheumatic mitral valve disease. They also reported a female predominance in MS cases. Similar gender distribution was also reported elsewhere.^22^-^24^

 The echocardiography measurements of the study patients revealed that the mean MVA was 1.29 ± 0.48 cm^2^. Comparison among patients with different grades of MS (mild, moderate and severe) with regard to the echocardiographic findings showed statistically significant/highly significant differences in TR grades, and RV and LA measurements.

 It was reported that TR frequently exists with MS. A moderate grade or higher of TR was shown in more than one-third of MS patients.^25^ TR was considered a complication and indicator of severity of MS.^25^ Accordingly, in our study, more cases of grade II TR were found in the moderate and severe MS groups.

 The present study showed that RV size and function, as assessed by TAPSE measurements, were significantly different among grades of MS, with the worst values seen in patients with severe MS. Actually, the RV dysfunction occurring in MS is attributed to LA hypertension with subsequent chronic pulmonary venous congestion and increased RV afterload. It was also suggested that depression of right myocardial function could be the sequel of direct RV rheumatic involvement, resulting in inflammation and necrosis of the myocytes, and further fibrosis and calcification.^26^ Our findings are congruent with the study by Ahmed et al,^27^ who reported that RV systolic function was significantly different between patients with different grades of MS.

 Significant changes in LA measurements among different grades of MS, which were found in our study, is consistent with data from previous studies. In the study by Ahmed and Awan,^28^ LA enlargement was observed in cases of MS, and the difference was according to the grade of MS. LA enlargement was reported to be the first sign of severity of MS.^25^

 With regard to the primary outcome of this study, MVA showed a statistically highly significant positive correlation with LA and RV function, and statistically significant/ highly significant negative correlation with their dimensions. Re-analysis of patients with isolated MS revealed the same significant association. The association between MVA and LA function was re-analysed in the current study using a ROC curve for assessment of LA function in predicting significant MS (moderate and severe MS). The AUC was 0.813, denoting good performance, with a statistically highly significant p-value. Moreover, MVA was found to be a predictor of LA function during the regression analysis (p = 0.000).

 The association between the MVA and LA size and function was in harmony with the pathophysiological changes, since the narrower the MVA, the higher the impact on the LA function and the more compensatory dilatation of the LA chamber. A few studies correlated MVA with echocardiographic findings. In agreement with our findings, Shojaeifard et al.,^29^ using a different parameter for LA function, peak LA longitudinal strain (PALS), demonstrated a significant correlation between the PALS measurements and MVA.

 Zulfa et al.^24^ tested the correlation between MVA and RV function as measured by TAPSE. They found a positive linear relationship between both, which is in harmony with the present study findings. This was also found in the study by Inci et al.^30^ This significant association suits the previously described reflection of MS on the RV function. The study of Iqbal et al.^23^ reported a weak correlation between LA size and MVA with an insignificant p-value, which is contradictory to our findings.

 With regard to the secondary outcome in our study, the ECG findings demonstrated that 47 patients were in sinus rhythm, while the remaining 34 were in AF (42%). Our figures are in the range that was previously described by Sahin et al.^31^ They reported that AF occurred in 40–75% of patients with MS.

 The present study showed that a higher percentage of patients with severe MS was in AF (58.1%). However, the difference between patients of both categories in the distribution of different grades of MS was not significant (p = 0.06). Patients in AF had statistically significantly higher LA areas and volumes, and statistically significantly lower LA function (p = 0.003).

 Within the same context, Shojaeifard et al.^29^ revealed that LA function, as measured by PALS, was significantly lower in AF patients than in those with MS in sinus rhythm. Nikdoust et al.^32^ evaluated LA function using a different parameter, early diastolic strain. They also demonstrated lower function in patients with AF. Caso et al.^33^ reported a significant decline in atrial strain measurements in AF patients.

 The relationship between the existence of AF and LA size was described in previous studies. In the study of Shrestha et al.,^34^ the majority of patients with AF showed LA size ≥ 4.0 cm, with a mean LA size of 4.6 cm. Bratt et al.^35^ demonstrated that LA volume was an independent predictor of the AF status.

## Strength and limitations

The strength of this work is that it is one of the few studies assessing the correlation between MVA and LA function. However, the relatively small sample size is a limitation. Also, LA volume assessment by 2D echocardiography is limited by significant geometric assumptions and low reproducibility because of diverging position and orientation of imaging planes. However, in this study, we excluded a large number of cases due to poor echogenic window of the LA, which could affect the validity of the main result. While 3D echocardiography and cardiac magnetic resonance imaging can undoubtedly increase the accuracy of determining the LA size, these techniques are not commonly available. Furthermore, multicentric, largescale studies using advanced echocardiographic procedures are recommended.

## Conclusion

There was a positive correlation between LA function and MVA in rheumatic MS patients. AF was associated with the severity of MS. 
